# Synthesis and Characterization of Novel Purpurinimides as Photosensitizers for Photodynamic Therapy

**DOI:** 10.3390/ijms15058091

**Published:** 2014-05-08

**Authors:** Bing Cun Cui, Il Yoon, Jia Zhu Li, Woo Kyoung Lee, Young Key Shim

**Affiliations:** 1 College of Medicine, Hubei Polytechnic University, Huangshi 435003, China; E-Mail: cuibc928@hotmail.com; 2 Photodynamic Therapy (PDT) Research Institute, School of Nano System Engineering, Inje University, Gimhae 621-749, Korea; 3 College of Chemistry and Chemical Engineering, Yantai University, Yantai 264005, China; E-Mail: jiazhu82@163.com

**Keywords:** purpurinimide, methyl pheophorbide *a*, photosensitizer, photodynamic therapy, singlet oxygen photogeneration, hydrophobicity parameter

## Abstract

A series of novel purpurinimides with long wavelength absorption were designed and synthesized to develop novel and potential photosensitizers. These compounds were investigated through reduction, oxidation, rearrangement reaction and amidation reactions of methyl pheophorbide *a*. They demonstrated a considerable bathochromic shift of the major absorption band in the red region of the optical spectrum (695–704 nm). Newly synthesized purpurinimides were screened for their antitumor activities, and showed higher photodynamic efficiency against A549 cell lines as compared to purpurin-18 methyl ester. The results revealed the novel purpurinimides could be potential photosensitizers.

## 1. Introduction

Photodynamic therapy (PDT) is a relatively new cancer modality that uses light, photosensitizers (PSs), and oxygen for the treatment of various forms of cancer by photodynamic action [1–3]. The PDT treatment derives great promise from the dual selectivity that is produced by both a preferential uptake of the drug by the diseased tissue and the restriction of carefully regulated light absorption onto the specific sites [4]. Regulatory approvals for the clinical use of PSs and PDT now exist in many countries around the world for treating cancers of the head and neck, brain, lung, pancreas, intraperitoneal cavity, breast, prostate and skin [5–7].

Chlorophyll (a natural dye) derivatives and related systems have been used as PSs in cancer phototherapy. Chlorophylls exhibit photophysical properties similar to porphyrin systems; the Q_y_ bands of chlorins is normally red-shifted to 20–30 nm and has a 10 times greater absorption intensity compared to porphyrins, which make chlorin-containing systems better candidates for PDT [8].

Purpurinimides, derived from chlorophyll-a, are tumor-avid PSs and show a strong absorption in the near IR region with a high singlet oxygen (^1^O_2_) producing efficiency, a key cytotoxic agent in PDT application [9]. PSs with long wavelength absorption should exhibit deeper tissue penetration, which is very useful to treat large and deeply seated tumors [10–12]. Therefore, the synthesis of novel purpurinimides has become the focus in PDT studies.

**Scheme 1.**
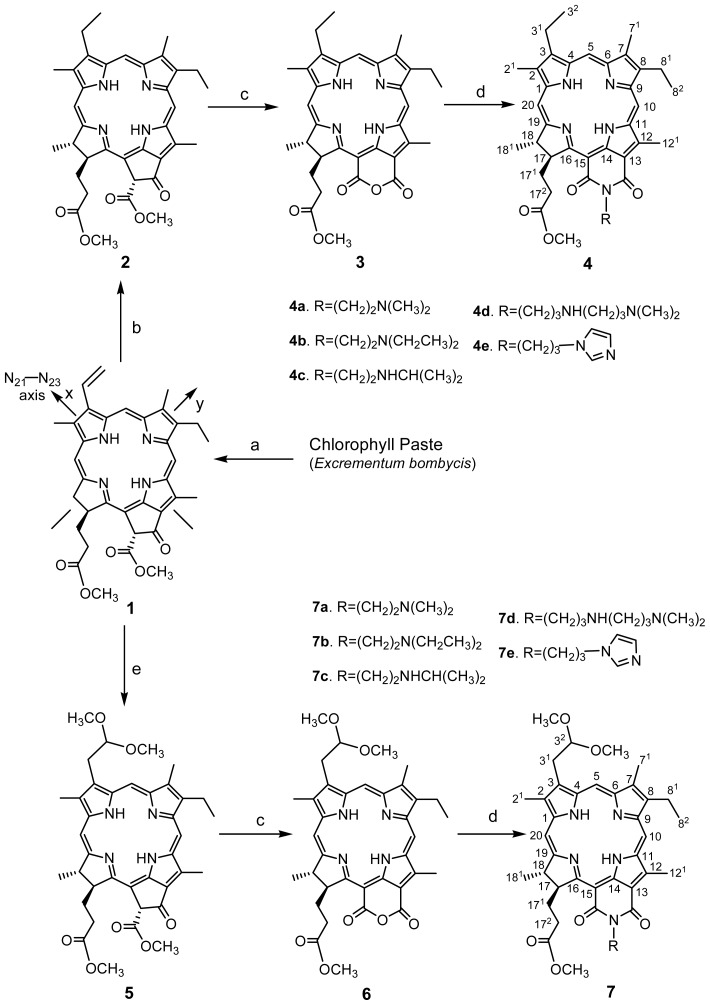
Synthesis of purpurin-18-*N*-aminoimides. Reagents and conditions. (**a**) 5% H_2_SO_4_/methanol, room temperature (rt); (**b**) (**i**) Zn(OAc)_2_/methanol, (**ii**) H_2_, Pd/C, tetrahydrofuran (THF), (**iii**) trifluoroacetic acid (TFA); (**c**) KOH/1-propanol/air, rt; (**d**) corresponding amine, toluene, reflux; and (**e**) Ti(NO_3_)_3_/methanol, rt.

For chlorophyll derivatives, the Q_y_ bands were strongly affected by introduced different substituent groups to Q_y_ axis (N_21_–N_23_, see Scheme 1). In continuation with our earlier efforts on PS design [13], it was thought worthwhile to synthesize new purpurinimide derivatives by incorporating the essential structural features of the above-mentioned potential cytotoxic drugs in order to obtain synergistic effects, which we report herein.

In the present work, we report the synthesis, structural characterization and biological evaluation of a series of novel purpurinimides through reduction, oxidation and rearrangement reactions followed by amidation reaction of methyl pheophorbide *a* (MPa).

## 2. Results and Discussion

### 2.1. Synthesis and Characterization

In our quest to compare the effect of imide analogs for quantitative structure-activity relationship (QSAR) studies [14], several attempts were made to convert the 3-vinyl group into the ethyl group and dimethyloxyethyl group by reduction and oxidation reaction.

The synthetic strategies adopted for the target compounds are shown in Scheme 1. MPa **1**, as an important starting material, was isolated from chlorophyll paste (*Excrementum bombycis*). The vinyl group at 3-position of MPa **1** was selectively hydrogenated by using Pd/C as a catalyst to yield *meso*-pheophorbide *a* **2**, which was converted to mesopurpurin-18 methyl ester **3** via air oxidation. Reaction of MPa **1** with Ti(NO_3_)_3_ at 0 °C produced the intermediate adduct **5** in high yield. Following the same procedure, 3-(2,2-dimethoxyethyl)-3-devinyl-purpurin-18 methyl ester **6** was obtained in 40% yield. From the reaction between two key intermediates **3** and **6**, and the corresponding amines, (**a**) *N*,*N*-dimethyl ethylamine; (**b**) *N*,*N*-diethyl ethylamine; (**c**) *N*-isopropyl ethylamine; (**d**) *N*,*N*-dimethylpropyl ethylamine; and (**e**) imidazolyl propylamine, successfully afforded the final purpurinimides **4a**–**4e** and **7a**–**7e** in excellent yield.

**Figure 1.**
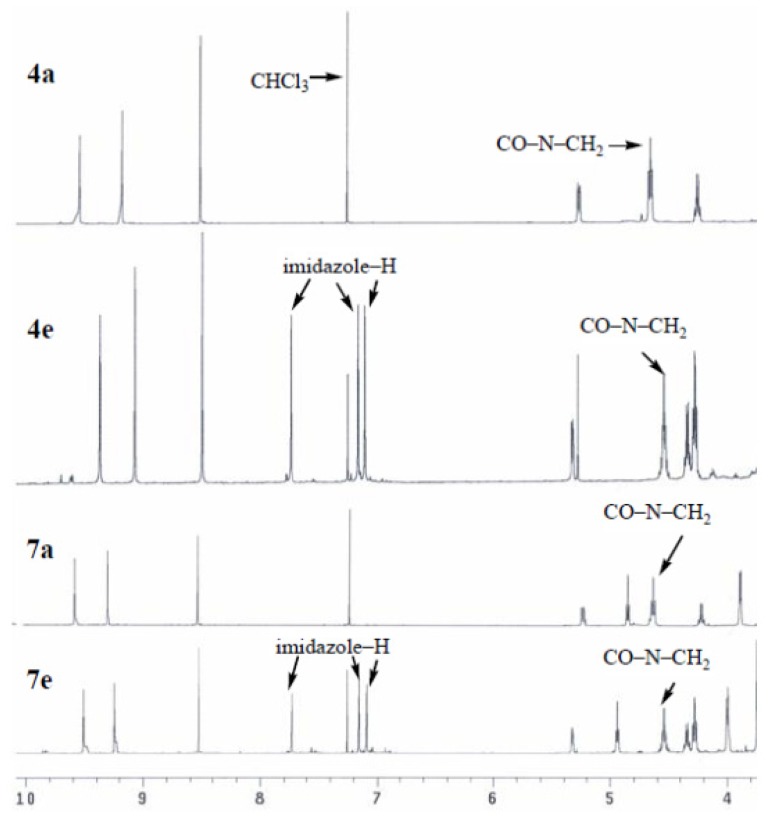
The comparative ^1^H NMR spectra (CDCl_3_, 500 Hz) in the region of δ 4.0–10.0 ppm of purpurinimides **4a**, **4e**, **7a** and **7e**.

All the purpurinimide compounds were successfully characterized by a combination analysis of ^1^H NMR and UV-vis spectroscopies, and elemental analysis. The structures of these novel purpurinimides were confirmed by ^1^H NMR spectroscopy (Figure 1). Compared to the mesopurpurin-18 methyl ester **3** the ^1^H NMR spectra of **4a**–**4e** showed each triplet at δ 4.54, 4.72, 4.58, 4.66 and 4.56 ppm for the protons of CO–N–CH_2_–, respectively. The imidazole protons of compound **4e** have three singlet signals at δ 7.73, 7.15 and 7.10 ppm. In compounds **4a** and **4d**, N–(CH_3_)_2_ protons appear as a singlet at δ 2.73 and 1.99 ppm, respectively. And compound **4b** shows a triplet at δ 1.27 ppm for the protons of *N*-(CH_2_CH_3_)_2_. Along with the proton signals in the chlorin macrocycle, signals of 3-(2,2-dimethoxyethyl) protons for 3-(2,2-dimethoxyethyl)-3-devinyl-purpurinimides were shown in δ 3.46 or 3.47 as a singlet. The ^1^H NMR spectra of **7a**–**7e** showed each triplet at δ 4.55, 4.74, 4.37, 4.84 and 4.58 ppm for the protons of CO–N–CH_2_–, respectively. The imidazole protons of compound **7e** reveal three singlet signals at δ 7.73, 7.16 and 7.09 ppm. In compounds **7a**, N–(CH_3_)_2_ protons appear as a singlet at δ 2.78 ppm. Purpurinimide **7b** shows a triplet at δ 1.44 ppm for the protons of N–(CH_2_CH_3_)_2_.

**Figure 2.**
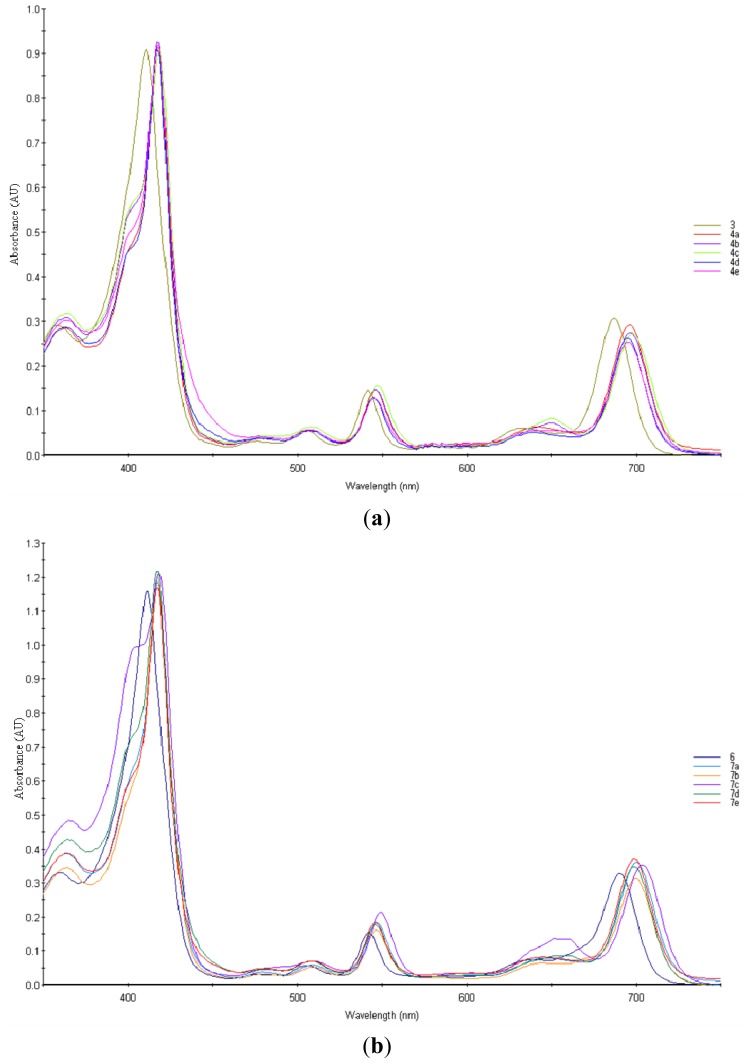
Electronic absorption spectra of purpurinimides for (**a**) **3**, **4a**–**4e**; and (**b**) **6**, **7a**–**7e** in CH_2_Cl_2_.

The spectroscopic properties of the purpurinimides in dichloromethane are shown in Figure 2 and summarized in Table 1. In the electronic absorption spectra, the long wavelength bands (Q_y_ band) of mesopurpurinimides **4a**–**4e** were observed in the range of 694–698 nm, compared with mesopurpurin-18 methyl ester **3** (686.7 nm), these mesopurpurinimides show a bathochromic shift of the Q_y_ band. The electronic spectrum of 3-(2,2-dimethoxyethyl)-3-devinyl-purpurinimides shows a bathochromic shift of the Q_y_ bands from 690.3 (compound **6**) to 700.1 (compound **7a**), 699.6 (compound **7b**), 703.6 (compound **7c**), 698.9 (compound **7d**) and 698.8 nm (compound **7e**). These purpurinimides had the “ideal” photochemical properties required for an effective PDT agent.

**Figure 3.**
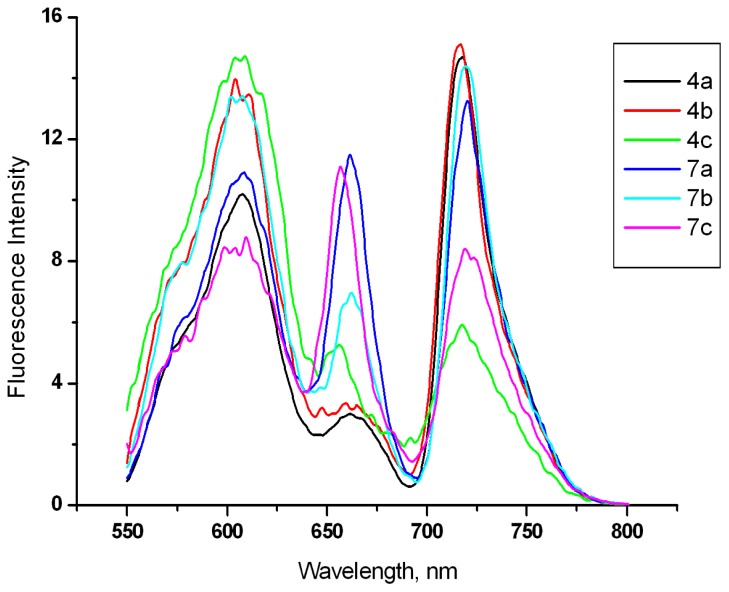
Fluorescence spectra of purpurinimides **4a**–**4c** and **7a**–**7c** (1.0 × 10^−4^ M) in dimethylsulfoxide (DMSO).

Fluorescence (emission) spectra of purpurinimides **4a**–**4c** and **7a**–**7c** in dimethylsulfoxide (DMSO) are shown in Figure 3. For all measurements the excitation wavelength was 530 nm. Three broad emission bands were observed (for **4a**, 550–644, 645–692, and 693–800 nm) with their emission maxima at 718 (**4a**), 717 (**4b**), 604 (**4c**), 720 (**7a**), 719 (**7b**), and 657 (**7c**) nm.

In addition, elemental analysis data for the purpurinimides reveal a good match between calculated and experimental values to the compounds, respectively.

### 2.2. In Vitro Study

**Figure 4.**
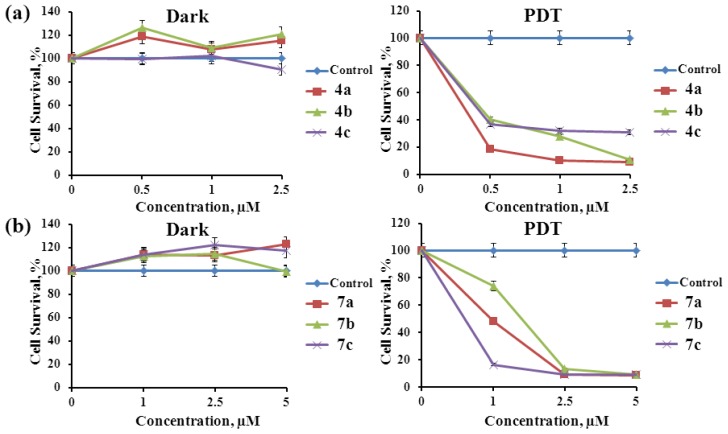
Comparative *in vitro* dark cytotoxicity and phototoxicity results for **4a**–**4c** and **7a**–**7c** at various drug doses (1–20 μM) against A549 cell lines by MTT (3-(4,5-dimethylthiazole-2-yl)-2,5-biphenyl tetrazolium bromide) assay at 12 h incubation after photoirradiation (670–710 nm, total light dose 2 J/cm^2^ for 15 min); (**a**) **4a**–**4c**; and (**b**) **7a**–**7c**. The data are expressed as mean of three experiments.

*In vitro* activity of the purpurinimides **4a**–**4c** and **7a**–**7c** was evaluated against A549 cell lines at various drug doses (1–10 or 1–20 μM) by MTT (3-(4,5-dimethylthiazole-2-yl)-2,5-biphenyl tetrazolium bromide) assay at 12 h incubation after photoirradiation (670–710 nm, total light dose 2 J/cm^2^ for 15 min). Compounds **4a**–**4c** showed no dark cytotoxicity until the highest concentration (in this case 10 μM, Figure 4a), otherwise, compounds **7a**–**7c** presented dark cytotoxicity more than 10 μM (Figure 4b). In all the compounds, upon photoirradiation, the cell viability was decreased consistent with increased concentration of drug dose (Figure 4). After photoirradiation, among all the purpurinimides, **4a** reveals the best photodynamic activity result (IC_50_ 0.28 μM, Table 2). The photodynamic activity is relatively higher in the order of **4a** > **4b** > **4c** > **7c** > **7a** > **7b**. These results suggest that the relatively different photodynamic activity results are significantly dependent on the functional groups at 3-position as well as various amino moieties on the chlorin macrocycle. Consequently, the novel purpurinimides exhibit excellent photodynamic activity, showing potential PSs for PDT.

### 2.3. Singlet Oxygen Study

**Figure 5.**
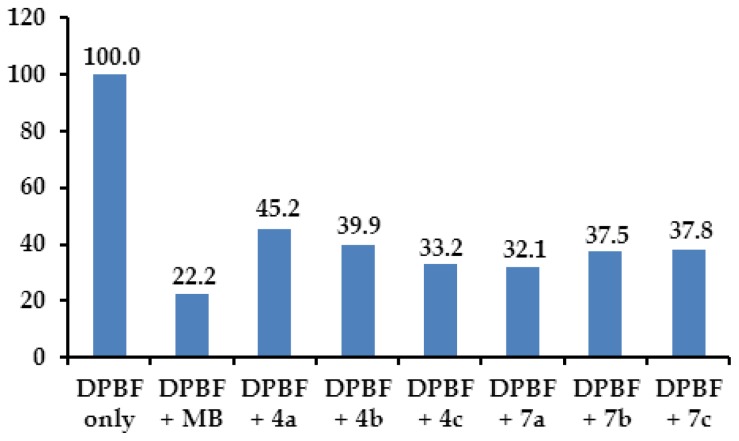
Comparative absorbance decay (%) of 1,3-diphenylisobenzofuran (DPBF) (50 μM in DMSO) at 418 nm after photoirradiation (total light dose 2 J/cm^2^, irradiation time 15 min) in the absence (control) and presence of 1 μM of MB (methylene blue), **4a**–**4c** and **7a**–**7c**. The data are expressed as mean of three experiments.

Figure 5 reveals relative difference of ^1^O_2_ photogeneration between the purpurinimides **4a**–**4c** and **7a**–**7c** using 1,3-diphenylisobenzofuran (DPBF) as a selective ^1^O_2_ acceptor [15]. Among the purpurinimides, **4c** and **7a** showed relatively higher ^1^O_2_ photogeneration, results which are slightly different from the *in vitro* results. Consequently, this result proves that the increased photodynamic activity attributed to the purpurinimides induced ^1^O_2_ photogeneration through cellular penetration and localization of the purpurinimides into the cells.

### 2.4. The Hydrophobicity Property Study

It is generally believed that the hydrophobicity parameter (logarithm of the partition coefficient between *n*-octanol and water; log *P*) is closely related to the cellular uptake. We applied a program module of the ACD/Labs software (version 12.01, Advanced Chemistry Development, Inc., Toronto, ON, Canada) to calculate the lipophilicity of the synthesized purpurinimides. Results are summarized in Table 3. All the purpurinimides have log *P* values up to log 5, which theoretically should be expected to give good PDT tumor response.

## 3. Experimental Section

### 3.1. General Methods

The ^1^H NMR spectra were recorded on a Varian-500 MHz spectrometer (Varian, Palo Alto, CA, USA). Chemical shifts are given as δ values using TMS as the internal standard and *J* values in Hz. The UV-visible spectra were recorded on S-3100 spectrophotometer (Scinco, Seoul, Korea) using dichloromethane as solvent. Elemental analysis was performed on Flash 2000 series of automatic elemental analyzer (Thermo Fisher Scientific, Milano, Italy) at Biohealth Products Research Center (BPRC), Inje University, Korea. Fluorescence spectra were obtained using a LS-50B Perkin Elmer luminescence spectrometer (Perkin Elmer, Waltham, MA, USA) at the Center for Research Facilities, Gyeongsang National University, Korea. The hydrophobicity parameter (logarithm of the partition coefficient between *n*-octanol and water; log *P*) was calculated on the basis of the purpurinimide structure using ACD/Labs software (version 12.01). Melting points (uncorrected) were measured on an Electrothermal IA9000s Series digital melting point apparatus. Thin-layer chromatography (TLC) was done on Merck silica gel 60 glass sheets (Cat. HX948839, layer thickness 0.25 mm) (Merck, Darmstadt, Germany). Column chromatography was performed over silica gel 60 (230–400 mesh) (Merck). In some cases, preparative TLC plates were also used for the purification (Analtech precoated silica gel GF glass plate, Cat. 01012, layer thickness 0.5 mm) (Merck). Materials obtained from commercial suppliers were used without further purification. MPa **1** [16], mesopurpurin-18 methyl ester **3** [9,16] and 3-(2,2-dimethoxyethyl)-3-devinyl-purpurin-18 methyl ester **6** [9,17] were prepared according to the literature procedures.

### 3.2. Preparation of Mesopurpurin-18-N-aminoimides

#### 3.2.1. General Procedure

In a typical experiment, mesopurpurin-18 methyl ester **3** (200 mg) and excess of each corresponding amine (0.15 mL) were dissolved in toluene (20 mL), and the mixture was refluxed under nitrogen atmosphere. After TLC showed complete consumption of purpurin-18 methyl ester, the mixture was cooled to room temperature, and then the solvent and excess amines were removed. The crude product was purified using silica column chromatography or preparative TLC plates with 10% methanol in dichloromethane to give corresponding purpurinimide **4a**, **4b**, **4c**, **4d** and **4e** as a purple solid, respectively.

#### 3.2.2. Characteristic Data for Mesopurpurin-18-*N*-(*N*,*N*-dimethyl)ethylimide **4a**

Yield: 96%. Mp: 99–101 °C. UV-vis in CH_2_Cl_2_, λ_max_ (nm, rel. intensity log ɛ), 417.3 (0.92), 476.8 (0.04), 508.0 (0.05), 545.7 (0.15), 695.8 (0.29). ^1^H NMR (500 MHz, CDCl_3_): δ 9.49 (s, 1H, 10H), 9.13 (s, 1H, 5H), 8.48 (s, 1H, 20H), 5.32 (m, 1H 17H), 4.72 (t, *J* = 7.0 Hz, 2H, N–*CH_2_*–CH_2_–N–(CH_3_)_2_), 4.33 (q, *J* = 7.5 Hz, 1H, 18H), 3.75 (s, 3H, 12^1^CH_3_), 3.72 (m, 2H, 3^1^CH_2_), 3.58 (s, 3H, 17^2^CO_2_CH_3_), 3.51 (m, 2H, 8^1^CH_2_), 3.22 (s, 3H, 2^1^CH_3_), 3.13 (s, 3H, 7^1^CH_3_), 2.73 (s, 6H, N–(CH_3_)_2_), 2.70, 2.42 and 1.96 (m, 6H, N–*CH_2_*–CH_2_–N–(CH_3_)_2_, 2 × 17^1^H and 2 × 17^2^H), 1.76 (d, *J* = 7.0 Hz, 3H, 18^1^CH_3_), 1.68 (t, *J* = 7.5 Hz, 3H, 8^2^CH_3_), 1.63 (t, *J* = 7.5 Hz, 3H, 3^2^CH_3_), 0.10 and −0.08 (each br s, 1H, 2NH). Anal. calcd. for C_38_H_46_N_6_O_4_: C, 70.13; H, 7.12; N, 12.91. Found: C, 70.18; H, 7.15; N, 12.92.

#### 3.2.3. Characteristic Data for Mesopurpurin-18-*N*-(*N*,*N*-diethyl)ethylimide **4b**

Yield: 95%. Mp: 118–120 °C. UV-vis in CH_2_Cl_2_, λ_max_ (nm, rel. intensity log ɛ), 417.4 (0.93), 477.1 (0.04), 507.6 (0.06), 546.0 (0.15), 649.6 (0.07), 696.4 (0.28). ^1^H NMR (500 MHz, CDCl_3_): δ 9.47 (s, 1H, 10H), 9.11 (s, 1H, 5H), 8.49 (s, 1H, 20H), 5.34 (m, 1H 17H), 4.58 (m, 2H, N–*CH_2_*–CH_2_–N–(CH_3_)_2_), 4.33 (q, *J* = 7.5 Hz, 1H, 18H), 3.74 (s, 3H, 12^1^CH_3_), 3.71 (q, *J* = 7.5 Hz, 2H, 3^1^CH_2_), 3.57 (s, 3H, 17^2^CO_2_CH_3_), 3.50 (m, 2H, 8^1^CH_2_), 3.22 (s, 3H, 2^1^CH_3_), 3.11 (s, 3H, 7^1^CH_3_), 2.89 (q, *J* = 7.0 Hz, 4H, N–(*CH_2_*CH_3_)_2_), 2.70, 2.40, 2.35 and 2.00 (m, 6H, N–CH_2_–*CH_2_*–N–(CH_2_CH_3_)_2_, 2 × 17^1^H and 2 × 17^2^H), 1.75 (d, *J* = 7.5 Hz, 3H, 18^1^CH_3_), 1.67 (t, *J* = 7.5 Hz, 3H, 8^2^CH_3_), 1.61 (t, *J* = 7.5 Hz, 3H, 3^2^CH_3_), 1.13 (t, *J* = 6.0 Hz, 6H, N–(CH_2_–*CH_3_*)_2_), 0.01 and −0.17 (each br s, 1H, 2NH). Anal. calcd. for C_40_H_50_N_6_O_4_: C, 70.77; H, 7.42; N, 12.38. Found: C, 70.80; H, 7.44; N, 12.39.

#### 3.2.4. Characteristic Data for Mesopurpurin-18-*N*-(*N*-isopropylamino)ethylimide **4c**

Yield: 96%. Mp: 100–102 °C. UV-vis in CH_2_Cl_2_, λ_max_ (nm, rel. intensity log ɛ), 418.3 (0.91), 477.0 (0.04), 508.2 (0.06), 5497.5 (0.16), 650.9 (0.08), 697.5 (0.27). ^1^H NMR (500 MHz, CDCl_3_): δ 9.30 (s, 1H, 10H), 9.04 (s, 1H, 5H), 8.47 (s, 1H, 20H), 5.32 (m, 1H 17H), 4.66 (m, 2H, N–*CH_2_*–CH_2_–NH–), 4.36 (q, *J* = 7.5 Hz, 1H, 18H), 3.68 (q, *J* = 7.5 Hz, 2H, 3^1^CH_2_), 3.64 (s, 3H, 12^1^CH_3_), 3.57 (s, 3H, 17^2^CO_2_CH_3_), 3.47 (m, 2H, 8^1^CH_2_), 3.21 (s, 3H, 2^1^CH_3_), 3.17 (m, *J* = 7.5 Hz, 1H, NH–*CH*–(CH_3_)_2_), 3.05 (s, 3H, 7^1^CH_3_), 2.71, 2.40, 2.06 and 1.97 (m, 6H, N–CH_2_–*CH_2_*–NH–, 2 × 17^1^H and 2 × 17^2^H), 1.75 (d, *J* = 7.5 Hz, 3H, 18^1^CH_3_), 1.66 (t, *J* = 7.5 Hz, 3H, 8^2^CH_3_), 1.56 (t, *J* = 7.5 Hz, 3H, 3^2^CH_3_), 1.18 (d, *J* = 6.0 Hz, 6H, NH–CH–(*CH_3_*)_2_), −0.03 and −0.17 (each br s, 1H, 2NH). Anal. calcd. for C_39_H_48_N_6_O_4_: C, 70.46; H, 7.28; N, 12.64. Found: C, 70.50; H, 7.29; N, 12.67.

#### 3.2.5. Characteristic Data for Mesopurpurin-18-*N*-(*N*,*N*-dimethylpropylamino)propylimide **4d**

Yield: 97%. Mp: 105–107 °C. UV-vis in CH_2_Cl_2_, λ_max_ (nm, rel. intensity log ɛ), 417.3 (0.91), 476.8 (0.04), 507.6 (0.06), 544.8 (0.13), 638.0 (0.05), 694.9 (0.26). ^1^H NMR (500 MHz, CDCl_3_): δ 9.44 (s, 1H, 10H), 9.10 (s, 1H, 5H), 8.47 (s, 1H, 20H), 5.26 (m, 1H 17H), 4.56 (m, 4H, N–*CH_2_*–CH_2_–*CH_2_*–), 4.32 (q, *J* = 7.5 Hz, 1H, 18H), 3.71 (s, 3H, 12^1^CH_3_), 3.70 (m, 2H, 3^1^CH_2_), 3.54 (s, 3H, 17^2^CO_2_CH_3_), 3.49 (m, 2H, 8^1^CH_2_), 3.22 (s, 3H, 2^1^CH_3_), 3.11 (s, 3H, 7^1^CH_3_), 2.95 (t, *J* = 7.0 Hz, 2H, N–CH_2_–CH_2_–CH_2_–NH–CH_2_–CH_2_–*CH_2_*–N–(CH_3_)), 2.72–2.64, 2.52–2.28, 2.21–2.15 (m, 8H, N–CH_2_–CH_2_–CH_2_–NH–*CH_2_*–*CH_2_*–CH_2_–N–(CH_3_), 2 × 17^1^H and 2 × 17^2^H), 1.99 (s, 6H, N–(CH_3_)_2_), 1.75 (d, *J* = 7.0 Hz, 3H, 18^1^CH_3_), 1.68 (t, *J* = 7.5 Hz, 3H, 8^2^CH_3_), 1.64 (m, 2H, N–CH_2_–*CH_2_*–CH_2_–NH–CH_2_–*CH_2_*–CH_2_–N–(CH_3_)), 1.62 (t, *J* = 7.0 Hz, 3H, 3^2^CH_3_), 0.20, 0.08 (each br s, 1H, 2NH). Anal. calcd. for C_42_H_55_N_7_O_4_: C, 69.88; H, 7.68; N, 13.58. Found: C, 69.92; H, 7.71; N, 13.56.

#### 3.2.6. Characteristic Data for Mesopurpurin-18-*N*-(imidazolyl)propylimide **4e**

Yield: 96%. Mp: 84–86 °C. UV-vis in CH_2_Cl_2_, λ_max_ (nm, rel. intensity log ɛ), 417.4 (0.92), 478.1 (0.04), 508.0 (0.06), 545.3 (0.13), 638.2 (0.06), 695.2 (0.25). ^1^H NMR (500 MHz, CDCl_3_): δ 9.36 (s, 1H, 10H), 9.06 (s, 1H, 5H), 8.48 (s, 1H, 20H), 7.73, 7.15, 7.10 (s, 3H, imidazole–H), 5.31 (m, 1H 17H), 4.34 (q, *J* = 7.0 Hz, 1H, 18H), 4.54, 4.27 (each m, 4H, N–*CH_2_*–CH_2_–*CH_2_*–), 3.69 (q, *J* = 7.5 Hz, 2H, 3^1^CH_2_), 3.68 (s, 3H, 12^1^CH_3_), 3.54 (s, 3H, 17^2^CO_2_CH_3_), 3.48 (m, 2H, 8^1^CH_2_), 3.21 (s, 3H, 2^1^CH_3_), 3.06 (s, 3H, 7^1^CH_3_), 2.71, 2.50, 2.36 and 2.02 (m, 6H, N–CH_2_–*CH_2_*–CH_2_–, 2 × 17^1^H and 2 × 17^2^H), 1.78 (d, *J* = 7.5 Hz, 3H, 18^1^CH_3_), 1.67 (t, *J* = 7.5 Hz, 3H, 8^2^CH_3_), 1.59 (t, *J* = 7.5 Hz, 3H, 3^2^CH_3_), 0.02 and −0.13 (each br s, 1H, 2NH). Anal. calcd. for C_40_H_45_N_7_O_4_: C, 69.85; H, 6.59; N, 14.25. Found: C, 69.88; H, 6.61; N, 14.26.

### 3.3. Preparation of 3-(2,2-Dimethoxyethyl)-3-devinyl-purpurin-18-N-aminoimides

#### 3.3.1. General Procedure

In a typical experiment, 3-(2,2-dimethoxyethyl)-3-devinyl-purpurin-18 methyl ester **6** (200 mg) and excess of each corresponding amine (0.15 mL) were dissolved in toluene (20 mL), and the mixture was refluxed under nitrogen atmosphere. After TLC showed complete consumption of purpurin-18 methyl ester, the mixture was cooled to room temperature, and then the solvent and excess amines were removed. The crude product was purified using silica column chromatography or preparative TLC plates with 10% methanol in dichloromethane to give corresponding purpurinimide **7a**, **7b**, **7c**, **7d** and **7e** as a purple solid, respectively.

#### 3.3.2. Characteristic Data for 3-(2,2-Dimethoxyethyl)-3-devinyl-purpurin-18-*N*-(*N*,*N*-dimethyl)ethyl-imide **7a**

Yield: 96%. Mp: 10–103 °C. UV-vis in CH_2_Cl_2_, λ_max_ (nm, rel. intensity log ɛ), 417.4 (1.19), 509.3 (0.06), 546.9 (0.18), 648.1 (0.08), 700.1 (0.36). ^1^H NMR (500 MHz, CDCl_3_): δ 9.54 (s, 1H, 10H), 9.26 (s, 1H, 5H), 8.52 (s, 1H, 20H), 5.33 (m, 1H 17H), 4.95 (t, *J* = 5.5 Hz, 1H, 3^2^H), 4.74 (t, *J* = 7.0 Hz, 2H, N–*CH_2_*–CH_2_–N–(CH_3_)_2_), 4.34 (q, *J* = 7.5 Hz, 1H, 18H), 4.02 (d, *J* = 5.0 Hz, 2H, 3^1^CH_2_), 3.76 (s, 3H, 12^1^CH_3_), 3.62 (q, *J* = 8.0 Hz, 2H, 8^1^CH_2_), 3.58 (s, 3H, 17^2^CO_2_CH_3_), 3.46 (s, 6H, 3^2^(OCH_3_)_2_), 3.27 (s, 3H, 2^1^CH_3_), 3.15 (s, 3H, 7^1^CH_3_), 2.78 (s, 6H, N–(CH_3_)_2_), 2.70, 2.40 and 1.97 (m, 6H, N–CH_2_–*CH_2_*–N–(CH_3_)_2_, 2 × 17^1^H and 2 × 17^2^H), 1.77 (d, *J* = 7.5 Hz, 3H, 18^1^CH_3_), 1.65 (t, *J* = 7.5 Hz, 3H, 8^2^CH_3_), 0.04 and −0.08 (each br s, 1H, 2NH). Anal. calcd. for C_40_H_50_N_6_O_6_: C, 67.58; H, 7.09; N, 11.82. Found: C, 67.60; H, 7.11; N, 11.85.

#### 3.3.3. Characteristic Data for 3-(2,2-Dimethoxyethyl)-3-devinyl-purpurin-18-*N*-(*N*,*N*-diethyl)ethyl-imide **7b**

Yield: 96%. Mp: 105–107 °C. UV-vis in CH_2_Cl_2_, λ_max_ (nm, rel. intensity log ɛ), 417.2 (1.19), 509.2 (0.06), 546.5 (0.16), 649.2 (0.08), 699.6 (0.31). ^1^H NMR (500 MHz, CDCl_3_): δ 9.50 (s, 1H, 10H), 9.24 (s, 1H, 5H), 8.52 (s, 1H, 20H), 5.27 (m, 1H, 17H), 4.94 (t, *J* = 5.5 Hz, 1H, 3^2^H)), 4.37 (3, 3H, N–*CH_2_*–CH_2_–N–(CH_2_CH_3_) and 18H), 4.01 (d, *J* = 5.5 Hz, 2H, 3^1^CH_2_), 3.73 (s, 3H, 12^1^CH_3_), 3.61 (q, *J* = 8.0 Hz, 2H, 8^1^CH_2_), 3.57 (s, 3H, 17^2^CO_2_CH_3_), 3.47 (s, 6H, 3^2^(OCH_3_)_2_), 3.27 (s, 3H, 2^1^CH_3_), 3.14 (s, 3H, 7^1^CH_3_), 2.90 (q, *J* = 7.0 Hz, 4H, N–(*CH_2_*CH_3_)_2_), 2.71, 2.40 and 2.00 (m, 6H, N–CH_2_–*CH_2_*–N–(CH_2_CH_3_)_2_, 2 × 17^1^H and 2 × 17^2^H), 1.81 (d, *J* = 7.0 Hz, 3H, 18^1^CH_3_), 1.64 (t, *J* = 7.5 Hz, 3H, 8^2^CH_3_), 1.44 (t, *J* = 6.0 Hz, 6H, N–(CH_2_–*CH_3_*)_2_), 0.13 and 0.08 (each br s, 1H, 2NH). Anal. calcd. for C_42_H_54_N_6_O_6_: C, 68.27; H, 7.37; N, 11.37. Found: C, 68.30; H, 7.39; N, 11.40.

#### 3.3.4. Characteristic Data for 3-(2,2-Dimethoxyethyl)-3-devinyl-purpurin-18-*N*-(*N*-isopropylamino)-ethylimide **7c**

Yield: 95%. Mp: 128–130 °C. UV-vis in CH_2_Cl_2_, λ_max_ (nm, rel. intensity log ɛ), 418.1 (1.21), 509.4 (0.07), 549.2 (0.21), 651.7 (0.14), 703.6 (0.35). ^1^H NMR (500 MHz, CDCl_3_): δ 9.07 (s, 1H, 10H), 8.78 (s, 1H, 5H), 8.48 (s, 1H, 20H), 5.21 (m, 1H 17H), 4.93 (t, *J* = 5.5 Hz, 1H, 3^2^H), 4.84 (m, 2H, N–*CH_2_*–CH_2_–NH–), 4.31 (q, *J* = 7.5 Hz, 1H, 18H), 4.00 (d, *J* = 5.5 Hz, 2H, 3^1^CH_2_), 3.67 (q, *J* = 6.5 Hz, 2H, 8^1^CH_2_), 3.55 (s, 3H, 17^2^CO_2_CH_3_), 3.47 (s, 6H, 3^2^(OCH_3_)_2_), 3.35 (m, *J* = 7.5 Hz, 1H, NH–*CH*–(CH_3_)_2_), 3.27 (s, 3H, 2^1^CH_3_), 2.97 (s, 3H, 7^1^CH_3_), 2.70, 2.37, 2.06 and 1.92 (m, 6H, N–CH_2_–*CH_2_*–NH–, 2 × 17^1^H and 2 × 17^2^H), 1.82 (d, *J* = 7.0 Hz, 3H, 18^1^CH_3_), 1.52 (d, *J* = 6.0 Hz, 6H, NH–CH–(*CH_3_*)_2_), 1.41 (t, *J* = 7.5 Hz, 3H, 8^2^CH_3_), 0.88 and −0.23 (each br s, 1H, 2NH). Anal. calcd. for C_41_H_52_N_6_O_6_: C, 67.93; H, 7.23; N, 11.59. Found: C, 69.97; H, 7.26; N, 11.62.

#### 3.3.5. Characteristic Data for 3-(2,2-Dimethoxyethyl)-3-devinyl-purpurin-18-*N*-(*N*,*N*-dimethylpropyl-amino)propylimide **7d**

Yield: 98%. Mp: 96–98 °C. UV-vis in CH_2_Cl_2_, λ_max_ (nm, rel. intensity log ɛ), 417.5 (1.21), 508.2 (0.07), 546.2 (0.18), 661.0 (0.09), 698.9 (0.35). ^1^H NMR (500 MHz, CDCl_3_): δ 9.37 (s, 1H, 10H), 9.15 (s, 1H, 5H), 8.48 (s, 1H, 20H), 5.27 (m, 1H 17H), 4.96 (t, *J* = 5.5 Hz, 1H, 3^2^H), 4.91, 4.58 (each t, 4H, N–*CH_2_*–CH_2_–*CH_2_*–), 4.34 (q, 1H, 18H), 4.03 (d, *J* = 5.5 Hz, 2H, 3^1^CH_2_), 3.65 (s, 3H, 12^1^CH_3_), 3.55 (s, 3H, 17^2^CO_2_CH_3_), 3.49 (m, 2H, 8^1^CH_2_), 3.46 (s, 6H, 3^2^(OCH_3_)_2_), 3.45 (s, 3H, 2^1^CH_3_), 3.10 (s, 3H, 7^1^CH_3_), 2.94 (t, *J* = 6.5 Hz, 2H, N–CH_2_–CH_2_–CH_2_–NH–CH_2_–CH_2_–*CH_2_*–N–(CH_3_)), 2.72–2.65, 2.44–2.30, 2.21–2.14 (m, 8H, N–CH_2_–CH_2_–CH_2_–NH–*CH_2_*–*CH_2_*–CH_2_–N–(CH_3_), 2 × 17^1^H and 2 × 17^2^H), 1.99 (s, 6H, N–(CH_3_)_2_), 1.76 (d, *J* = 7.5 Hz, 3H, 18^1^CH_3_), 1.66 (m, 2H, N–CH_2_–*CH_2_*–CH_2_–NH–CH_2_–*CH_2_*–CH_2_–N–(CH_3_)), 1.59 (t, *J* = 7.0 Hz, 3H, 8^2^CH_3_), 0.04 (br s, 1H, NH). Anal. calcd. for C_44_H_59_N_7_O_6_: C, 67.58; H, 7.60; N, 12.54. Found: C, 67.61; H, 7.62; N, 12.55.

#### 3.3.6. Characteristic Data for 3-(2,2-Dimethoxyethyl)-3-devinyl-purpurin-18-*N*-(imidazolyl)-propylimide **7e**

Yield: 97%. Mp: 87–89 °C. UV-vis in CH_2_Cl_2_, λ_max_ (nm, rel. intensity log ɛ), 417.4 (1.17), 509.1 (0.07), 545.9 (0.18), 645.0 (0.08), 698.8 (0.37). ^1^H NMR (500 MHz, CDCl_3_): δ 9.51 (s, 1H, 10H), 9.24 (s, 1H, 5H), 8.53 (s, 1H, 20H), 7.16, 7.09 (s, 3H, imidazole–H), 5.34 (m, 1H 17H), 4.94 (t, *J* = 5.5 Hz, 1H, 3^2^H), 4.55, 4.28 (each m, 4H, N–*CH_2_*–CH_2_–*CH_2_*–), 4.35 (q, *J* = 7.0 Hz, 1H, 18H), 4.00 (d, *J* = 5.5 Hz, 2H, 3^1^CH_2_), 3.75 (s, 3H, 12^1^CH_3_), 3.57 (q, *J* = 7.5 Hz, 2H, 8^1^CH_2_), 3.54 (s, 3H, 17^2^CO_2_CH_3_), 3.46 (s, 6H, 3^2^(OCH_3_)_2_), 3.27 (s, 3H, 2^1^CH_3_), 3.14 (s, 3H, 7^1^CH_3_), 2.73, 2.54, 2.09 and 2.01 (m, 6H, N–CH_2_–*CH_2_*–CH_2_–, 2 × 17^1^H and 2 × 17^2^H), 1.77 (d, *J* = 7.0 Hz, 3H, 18^1^CH_3_), 1.64 (t, *J* = 7.5 Hz, 3H, 8^2^CH_3_), 0.02 and −0.11 (each br s, 1H, 2NH). Anal. calcd. for C_42_H_49_N_7_O_6_: C, 67.45; H, 6.60; N, 13.11. Found: C, 67.48; H, 6.62; N, 13.13.

### 3.4. In Vitro Photosensitizing Efficacy

A549 cell lines were cultured at 37 °C in a humidified 5% CO_2_ incubator using RFMI 1640 growth medium supplemented with 10% fetal bovine serum and 1% penicillin/streptomycin. For phototoxicity studies, A549 cells were plated in 96-well plates at a density of 1 × 10^5^ cells/well. After 24 h of incubation, 100 μL of 1, 2, 5, 10 and 15 μM purpurinimides were added in each well, respectively. Plates were returned to the incubator for 24 h. And then the cells were replaced with fresh media and exposed to light (640–710 nm, total dose 2.0 J/cm^2^ for 15 min). Following illumination, the plates were incubated at 37 °C in the dark. After 3, 12 and 24 h incubations, MTT solution was added into each well and the absorbance was measured by fluorescence multi-detection reader (BioTek, Synergy HT, Winooski, VT, USA) at 450 nm. Each group consisted of 3 wells. The percentage cell survival was calculated by normalization with respect to the value for no PS treatment (control).

### 3.5. Measurement of Singlet Oxygen Photogeneration

1,3-Diphenylisobenzofuran (DPBF) was used as a selective ^1^O_2_ acceptor, which was bleached upon reaction with ^1^O_2_ [15]. Five sample solutions of DPBF in DMSO (50 μM) containing, respectively, DPBF only (50 μM, control sample), DPBF + methylene blue (MB) (1 μM), DPBF + **4a** (1 μM), DPBF + **4b** (1 μM), DPBF + **4c** (1 μM), DPBF + **7a** (1 μM), DPBF + **7b** (1 μM), DPBF + **7c** (1 μM) were prepared in dark. All the samples were placed in a 96-well plate and the container was covered with aluminum foil. The samples were irradiated (2 J/cm^2^) for 15 min. After irradiation, visible spectra of the sample solutions were measured spectrophotometrically. The normalized absorbances of DPBF at 418 nm in these samples were reported. The ^1^O_2_ photogeneration activities of MB, **4a**–**4c**, and **7a**–**7c** can be compared with the different absorbance decay of each sample relative to the DPBF control sample.

## 4. Conclusions

We described the synthesis of novel purpurinimides, mesopurpurin-18-*N*-aminoimides and 3-(2,2-dimethoxyethyl)-3-devinyl-purpurin-18-*N*-aminoimides with various amines (*N*,*N*-dimethyl ethylamine, *N*,*N*-diethyl ethylamine, *N*-isopropyl ethylamine, *N*,*N*-dimethylpropyl ethylamine and imidazolyl propylamine). The final desired purpurinimides were obtained in excellent yield. The purpurinimides were characterized by a combination analysis of ^1^H NMR, UV-vis and photoluminescence spectroscopies, and elemental analysis. ^1^H NMR spectroscopy confirms the structures of purpurinimides using ammonium formation after methylation, resulting in significant down field shift of the methyl proton signals. In the electronic absorption spectra, compared with starting materials, these purpurinimides show a bathochromic shift (8–13 nm) of the Q_y_ band, resulting in a long wavelength absorption (695–704 nm) which should be helpful for promoting deep light penetration into tumor tissue because of minimal light scattering. Fluorescence spectra present three broad emission bands at 550–800 nm range. Preliminary *in vitro* studies demonstrate that the new purpurinimides revealed excellent photodynamic efficacy (IC_50_ 0.28–1.27 μM at 12 h incubation time after photoirradiation), which corresponds the excellent ^1^O_2_ photogeneration of the purpurinimides. Among the purpurinimides, mesopurpurin-18-*N*-(*N*,*N*-dimethyl)ethylimide **4a** presents the best photodynamic activity result. The photodynamic activity is relatively higher in the order of **4a** > **4b** > **4c** > **7c** > **7a** > **7b**, results which are related to the hydrophobicity property (log *P*). For the purpurinimides to be potential candidates for PDT, further *in vivo* studies (e.g., pharmacokinetics and tissue distribution tests) are desirable, which is currently under investigation. This result could be useful for synthesis and development of new potential PSs as well as for understanding of QSAR study in PDT.

## Figures and Tables

**Table 1.** Absorption properties of the purpurinimides (**3**, **4a**–**4e**, **6**, **7a**–**7e**) in CH_2_Cl_2_.

Compound	Absorption λ_max_ (nm) (log ɛ) a			

	Soret	ΔSoret (Δɛ)	Q_y_	ΔQ_y_ (Δɛ)
**3**	410.8 (0.91)	0	686.7 (0.31)	0
**4a**	417.3 (0.92)	6.5 (0.01)	695.8 (0.29)	9.1 (−0.02)
**4b**	417.4 (0.93)	6.6 (0.02)	696.4 (0.28)	9.7 (−0.03)
**4c**	418.3 (0.91)	7.5 (0)	697.5 (0.27)	10.8 (−0.04)
**4d**	417.3 (0.91)	6.5 (0)	694.9 (0.26)	8.2 (−0.05)
**4e**	417.4 (0.92)	6.6 (0.01)	695.2 (0.25)	8.5 (−0.06)
**6**	411.5 (1.16)	0	690.3 (0.33)	0
**7a**	417.4 (1.19)	5.9 (0.03)	700.1 (0.36)	9.8 (0.03)
**7b**	417.2 (1.19)	5.7 (0.03)	699.6 (0.31)	9.3 (−0.02)
**7c**	418.1 (1.21)	6.6 (0.05)	703.6 (0.35)	13.3 (0.02)
**7d**	417.5 (1.21)	6.0 (0.05)	698.9 (0.35)	8.6 (0.02)
**7e**	417.4 (1.17)	5.9 (0.01)	698.8 (0.37)	8.5 (0.04)

a ΔSoret, ΔQ_y_ and Δɛ represent the change of the Soret band, Q_y_ band and absorbance intensity, respectively, between the substituted purpurinimides and corresponding starting materials.

**Table 2.** IC_50_ values of the purpurinimides against A549 cell lines at 12 h incubation time after photoirradiation.

Compound	4a	4b	4c	7a	7b	7c
IC_50_ a (μM)	0.28	0.37	0.40	0.95	1.27	0.70

a IC_50_ presents the half maximal (50%) inhibitory concentration of the compound.

**Table 3.** Hydrophobicity parameters (log *P*) of the purpurinimides calculated by means of computer software ACD/Labs (version 12.01).

Compound	Log *P*
**4a**	6.94 ± 1.62
**4b**	8.00 ± 1.62
**4c**	7.32 ± 1.62
**4d**	6.89 ± 1.63
**4e**	6.88 ± 1.62
**7a**	5.80 ± 1.64
**7b**	6.87 ± 1.64
**7c**	6.19 ± 1.64
**7d**	5.76 ± 1.65
**7e**	5.75 ± 1.64
